# First assessment of the eHealth Literacy Questionnaire (eHLQ) among French people with chronic kidney disease

**DOI:** 10.1186/s12882-026-04874-5

**Published:** 2026-06-09

**Authors:** Lucile Paris, Rémy Morello, Eve Calvar, Thierry Lobbedez, Valérie Châtelet

**Affiliations:** 1 Centre Universitaire des Maladies Rénales, CHU de Caen, Avenue de la Côte de Nacre, Caen, Cedex 9, 14 033 France; 2 U1086 INSERM – ANTICIPE – Centre Régional de Lutte Contre le Cancer, François Baclesse, Caen, France; 3 Plateforme de Méthodologie, CHU de Caen, Avenue de la Côte de Nacre, Niveau 3, CS 30001, Caen, Cedex 9, 14033 France; 4 Normandie Université, Unicaen, UFR de Médecine, 2 rue des Rochambelles, Caen, Cedex, 14032 France

**Keywords:** Chronic kidney disease, eHealth literacy, Social inequalities in health, Exploratory factor analysis

## Abstract

**Background:**

Social deprivation limits access to the kidney transplant waiting list. Possible mediators of this association could be health literacy (HL) and eHealth literacy (e-HL) levels. Digital health interventions have been developed to improve patient health information and education. Their development should be based on the e-HL level of the target population to meet their health needs. A tool is needed to measure the e-HL of patients with chronic kidney disease (CKD). The aim of this study was to assess the eHealth Literacy Questionnaire (eHLQ) in a French population with CKD.

**Methods:**

Patients with CKD were recruited from January to March 2023 to complete the eHLQ. Three other questionnaires were completed to assess HL and dependence in daily life. The analysis included item assessment, scale reliability and exploratory factor analysis (EFA).

**Results:**

A total of 371 patients completed the questionnaire. The analysis suggested a different structure, as the 4-factor EFA was the most meaningful and appropriate. The internal consistency was adequate for each scale. The highest score was obtained for scale 4, “Feel safe and in control” and the lowest for scale 1, “Using technology to process health information”.

**Conclusions:**

Our study suggests that a division of the eHLQ into 4 scales should better reflect e-HL among French patients with CKD. The eHLQ should be modified to improve its construct validity. A validated tool to evaluate e-HL in the population with CKD is essential to improve health equity in access to health education and healthcare.

**Supplementary Information:**

The online version contains supplementary material available at 10.1186/s12882-026-04874-5.

## Background

It is widely accepted that kidney transplantation is the best treatment for patients with end-stage kidney disease (ESKD) who have no medical contraindications and who are willing to be transplanted [1]. Internationally, ESKD patients in high-income countries have better access to kidney transplantation than patients from middle- and low-income countries [2]. At the national level, we showed, using the European Deprivation Index, that social deprivation was associated with restricted access to the kidney transplant waiting list [3] and to preemptive and living donor kidney transplantation [4]. A mediation analysis demonstrated that predialysis care partly explained the association between social deprivation and lower rates of early registration. However, other causal pathways for this association need to be explored [3].

Limited health literacy (HL) is common among patients with chronic kidney disease (CKD), particularly those from lower socioeconomic backgrounds or nonwhite minorities [5]. This is a matter of concern, as poor health literacy is associated with poorer adherence to medication, more hospital admissions and fewer referrals for kidney transplant assessment [6–9]. The level of HL could affect access to the waiting list for kidney transplantation [10] and, therefore, explain social disparities in access to the waiting list.

Digital health interventions have been developed to improve patient information and education. They can have a positive impact on access to the health care system and on patient outcomes [11–14]. Despite the development of these digital services, there is a lack of evidence that patients with CKD are able and willing to use them [15]. Therefore, an appropriate tool is needed to assess electronic health literacy (e-HL) to understand whether these “new technologies” suit patients’ needs and skills, which is essential to improve patients’ information and education [16]. In addition, a validated e-HL questionnaire for patients with CKD would allow the relationship between social deprivation and access to the waiting list to be explored and, subsequently, interventions to reduce health disparities to be implemented.

The eHLQ (eHealth Literacy Questionnaire) is a multidimensional questionnaire based on a well-defined e-HL framework [17]. Compared to previous e-HL tools [18], the eHLQ takes into account the recent changes in digital services in health care systems. It includes different scales to explore the broad concept of e-HL covering the knowledge and abilities of the person and the individual’s interaction with digital services in a functional connected health system [19]. The psychometric properties of the eHLQ have been found to be robust in previous studies from : Denmark (475 participants recruited in hospital, health centers, outpatient clinic, libraries and private sector workplaces), Australia (525 patients, mean age 57 years, 38.7% male, recruited in community health sites, 57.1% of whom had a chronic condition), Taiwan (420 patients, mean age 54.7 years, 61.7% male, Taiwanese and Mandarin speaking, recruited in outpatient departments of cardiology, nephrology, endocrinology and family medicine, 41.7% suffered from chronic kidney disease), and Sweden (236 participants, mean age 48 years, 44.5% male, recruited in primary healthcare centers and parents of hospitalised children) [19–23]. The objective of our study is to assess the structure of the eHLQ in a French population with CKD. In a further study, a confirmatory factor analysis will be performed to assess the validity of the eHLQ in this population. To the best of our knowledge, this is the first study focusing on the e-HL among a population with CKD.

## Methods

### Questionnaires

The eHLQ comprises 35 items (Q1–35) that measure 7 scales (S1–7), with 4 to 6 items per scale (Table 1). The items are scored on a Likert scale ranging from 1 (strongly disagree) to 4 (strongly agree). Scale scores are calculated by averaging the scores of the items in each scale, with equal weighting applied. This yields 7 scores, each ranging from 1 to 4. No overall eHLQ score is calculated.

Licence to use the French version of the eHLQ was obtained from Swinburne University, Australia, following a process of translation and back-translation conducted by Epstein et al. (REFLIS: Réseau Francophone de Littératie en Santé, not published).

The BHLS (Brief Health Literacy Screen) [24, 25] and the FCCHL (Functional, Communicative and Critical Health Literacy Scale) [26] were used to assess health literacy (Table SUP1, Table SUP2), while the HAQ (Health Assessment Questionnaire) [27] was administered to estimate patients’ dependence in daily life (Table SUP3).

**Table 1:** eHealth Literacy Questionnaire scale names and items

**1. Using technology to process health information/Utiliser les nouvelles technologies pour gérer des informations de santé**
I use technology to find…/J’utilise les nouvelles technologies pour obtenir…
I often use technology to understand…/J’utilise souvent les nouvelles technologies pour comprendre…
Technology helps me decide…/Les nouvelles technologies m’aident à faire les bons choix…
I use technology to share…/J’utilise les nouvelles technologies pour partager…
I use technology to organise…/J’utilise les nouvelles technologies pour gérer…
**2. Understanding of health concepts and language/Compréhension des concepts médicaux et du langage médical**
The information I have helps me…/Mes connaissances me permettent…
I have enough information to take part in… /J’ai suffisamment d’informations pour participer…
I understand medical results…/Je comprends mes résultats…
Overall, I understand how my body…/Dans l’ensemble, je comprends comment mon corps…
I use measurements about my body to…/J’utilise des données corporelles…
**3. Ability to actively engage with digital services/Capacité à s’investir activement avec les services numériques**
I know how to get…/Je sais comment utiliser…
I know how to make health technology…/Je sais utiliser les nouvelles technologies…
I can enter data…/Je suis capable de saisir des données…
I quickly learn how to…/J’apprends rapidement à me débrouiller…
I easily learn to use…/J’apprends facilement à me servir des…
**4. Feel safe and in control/Se sentir en sécurité et en maîtrise**
I am sure that my health data…/Je suis sûr(e) que mes données de santé…
My electronic health care data are being stored…/Mes données électroniques de santé sont stockées…
I have a clear understanding of…/Je comprends bien comment…
I am sure that only authorised people…/Je suis sûr(e) que seules les personnes autorisées…
I am confident that health care providers… /J’ai confiance dans le fait que les professionnels de santé…
**5. Motivated to engage with digital services/Motivation à s’investir dans les services numériques**
Technology makes me feel actively…/Les nouvelles technologies me donnent le sentiment d’être acteur…
I find technology helps me…/Je trouve que les nouvelles technologies m’aident…
I find I get better services…/Je trouve que je suis mieux pris en charge…
Technology improves my communication…/Les nouvelles technologies facilitent ma communication…
I find technology useful…/Je trouve que les nouvelles technologies sont utiles…
**6. Access to digital services that work/Accès à des services numériques qui fonctionnent**
Information about my health is always available…/Mes informations de santé sont accessibles…
My health care providers deliver services that I can access…/Mes professionnels de santé proposent des services auxquels je peux accéder…
My health data are available…/Je peux accéder à mes données de santé…
All the health technology I use works…/Toutes les nouvelles technologies de santé que j’utilise fonctionnent…
Most of my health care providers can be accessed…/La plupart de mes professionnels de santé peuvent être contactés…
I have access to health technology that…/J’ai accès à de nouvelles technologies de santé qui…
**7. Digital services that suit individual needs/Services numériques adaptés aux besoins individuels**
I find that digital services can adapt…/Je trouve que les dispositifs de santé connectée s’adaptent…
I find that digital health services seem to…/Je trouve que les dispositifs de santé connectés s’adaptent…
I find digital health services are provided to me in a way…/Les dispositifs de santé connectée me sont proposés d’une manière…
Digital health services provide me with easy ways…/Les dispositifs de santé connectée me proposent des solutions pratiques…

Response categories to all items: strongly disagree, disagree, agree, strongly agree. Items are truncated. The full list of items is available with the authors

### Study population

Patients were recruited from individuals who volunteered in the nephrology service at the University Hospital of Caen (Normandy) between January and March 2023. Eligible participants for this study were patients with chronic kidney disease (CKD) who were over 18 years old and able to read and understand French. Individuals with difficulties in reading, writing, or understanding French, as well as those with visual or cognitive disorders, were excluded from the survey.

Respondents provided informed consent and completed the questionnaire anonymously, using paper and pen. They could either fill out the questionnaire themselves or have it read to them by a caregiver. The target sample size was set at 350 respondents, based on the rule of “10 subjects per item” [28].

### Cognitive interviews

Cognitive interviews were conducted to assess content validity. A target of 30 patients was set to ensure that each item was evaluated by at least 4 patients, with a maximum of 5 items assessed per patient to minimize fatigue [29]. Patients were randomly recruited to maintain a representative proportion of individuals under each treatment modality (in-center hemodialysis, autonomous dialysis, transplantation, and non-dialysis patients).

During the interviews, we read the questionnaire aloud to the patients and recorded any reactions they expressed. We then reviewed in detail both the items that elicited reactions and the predefined items, ensuring that each item was evaluated by four patients. Patients were asked whether the wording of each item was clear and were encouraged to rephrase or explain its meaning. The goal was to verify that the wording of the items was both understandable and unambiguous.

Patients who underwent interviews were included in the psychometric analysis.

### Studied variables

Sociodemographic variables—such as age, gender, nationality, educational level, employment status, living area, and whether the patient lived alone—were collected. Additional variables included transplantation status (e.g., whether the patient was registered on the kidney transplantation waiting list) and treatment modality. Treatment modalities comprised transplantation, in-center dialysis, autonomous dialysis (including peritoneal dialysis (PD), home hemodialysis (HD), and self-care dialysis units), as well as non-dialysis-dependent chronic kidney disease (CKD) with a glomerular filtration rate (GFR) of less than 30 mL/min/1.73 m².

### Statistical analyses

Continuous variables are described using medians and the 1st and 3rd quartiles. For the eHLQ scales, means and standard deviations are also provided. Categorical variables are presented as frequencies and percentages. The Mann–Whitney U test, Kruskal–Wallis test, and Spearman’s correlation coefficients were used to examine the relationships between the scores of each eHLQ scale and respondent characteristics, as the variables did not follow a normal distribution according to Shapiro–Wilk tests.

The psychometric properties of the eHLQ were assessed based on classical test theory (CTT). Item quality was evaluated through acceptability and the presence of floor or ceiling effects. Item difficulty (mean score per item) and item discrimination (assessed via corrected item-total correlation) were also analysed [30]. Construct validity was evaluated using a scree plot, structural validity, and convergent and divergent validity. Structural validity was explored through a multitrait-multimethod (MTMM) approach [31, 32] and factor analysis, with items retained in each factor if their loadings were 0.30 or higher [33].

To assess convergent and divergent validity, only respondents with missing data for fewer than 5 FCCHL questions and no missing data for the BHLS or HAQ were included [29]. Moderate to strong correlations (ρ > 0.4) were expected between the 2nd scale of the eHLQ and the BHLS and FCCHL, as these scales all measure health literacy. Conversely, a negative correlation was expected between the HAQ and the eHLQ, as they assess distinct and theoretically opposite constructs.

Internal consistency was evaluated using Cronbach’s alpha with a 95% confidence interval for each scale. McDonald’s omega was used as a complementary indicator of internal consistency, as it provides a more robust estimate of reliability than Cronbach’s alpha under less restrictive assumptions. Unlike Cronbach’s alpha, which assumes tau-equivalence (i.e.equal factor loadings across items), omega takes into account the actual factor loadings derived from the measurement model and therefore better reflects the true proportion of variance attributable to the latent construct. Omega is particularly recommended when items show heterogeneous loadings or when multidimensionality is suspected, as is often the case in health-related questionnaires [34].

A supplementary index is available to clarify specific concepts related to questionnaire validation (see Supplementary Data).

### Missing data

Depending on the rate and pattern of missing data, an imputation of missing values for the eHLQ items was planned using a regularised iterative PCA-based method if the “missing at random” was plausible [35].

Statistical analyses were performed with R 4.2.0 (R Foundation for Statistical Computing) using psych and missMDA packages.

## Results

### Cognitive interviews

Overall, patients’ understanding of the items was similar to the items’ intents. The items were clearly understood by respondents, except for 8 items (Q13S1, Q25S1, Q4S3, Q14S4, Q2S5, Q3S6, Q23S6, Q34S6) whose wording was considered confusing by more than 50% of the interviewed patients. Most of the patients did not have a clear understanding of the terms covering health technology services and did not relate these terms well to their medical situation within the health care system.

### Participant characteristics

**Fig. 1:**
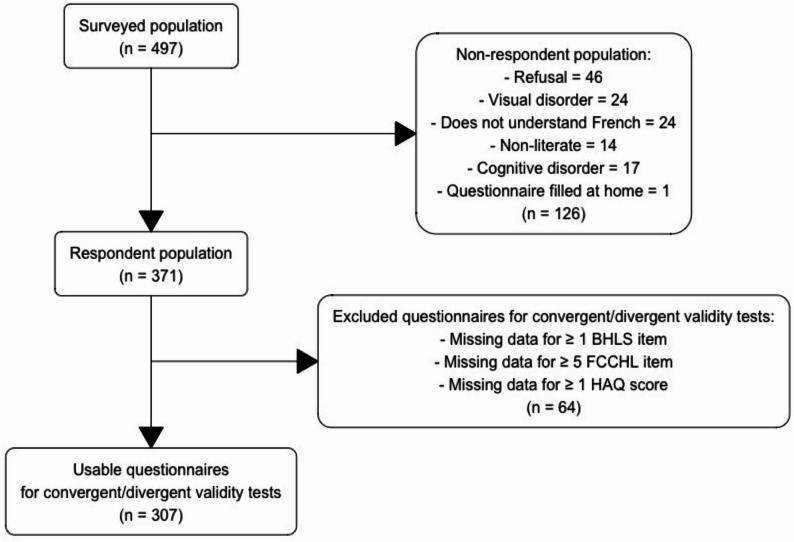
Flow chart of the study. BHLS: Brief Health Literacy Screen, FCCHL: Functional Communicative and Critical Health Literacy scale, HAQ: Health Assessment Questionnaire

A total of 371 patients responded to the questionnaire. Forty-six patients declined to complete the questionnaire for several reasons (of which 22 patients were unwilling, and 11 had no internet access or no use of digital services). The flow chart is displayed in Fig. 1.

Patient characteristics are displayed in Table 2. The median age was 64 years [18–93]. The sex ratio was (M/F) 243/127. Respondents were mostly from France (93%) and had a low level of education, with 65% who discontinued studying during high school or earlier. 30% of the respondents were transplanted, 26.7% were non-dialysis-dependent patients with CKD, and almost 44% were on dialysis (of which 20% were on autonomous dialysis).

**Table 2:** Sociodemographic characteristics of the respondent population

Variable	*N* = 371^1^
**Age**	64 (53, 72)
* Missing*	13
**Gender**	
* Male*	243 (66%)
* Female*	127 (34%)
* Missing*	1
**Nationality**	
* Europe (except France)*	5 (1.4%)
* Africa*	17 (4.8%)
* Asia*	2 (0.6%)
* France*	330 (93%)
* Missing*	17
**Education level**	
* High school or below*	235 (65%)
* Bachelor’s degree or college education*	125 (35%)
* Missing*	11
**Employment status**	
* Employed*	90 (24%)
* Unemployed*	42 (11%)
* Student*	4 (1.1%)
* Retired*	207 (56%)
* Others*	28 (7.5%)
**Living area**	
* City centre*	88 (24%)
* Suburban area*	98 (27%)
* Countryside*	178 (49%)
* Missing*	7
**Living alone**	
* Lives with someone*	274 (75%)
* Lives alone*	89 (25%)
* Missing*	8
**Treatment modality**	
* CKD*	94 (26.7%)
* Autonomous dialysis*	70 (19.9%)
* In-centre dialysis*	84 (23.9%)
* Kidney transplantation*	104 (29.5%)
* Missing*	19
**Transplantation status**	
* Transplanted*	105 (36%)
* Registered on the waiting list*	50 (17%)
* Transplantation assessment*	22 (7.5%)
* Temporary contraindication*	12 (4.1%)
* Not registered*	80 (27%)
* Does not feel concerned about transplantation*	24 (8.2%)
* Missing*	78

^1^ Continuous: median (1st quartile, 3rd quartile),Categorical: Frequency (%), CKD: Chronic kidney disease

### Missing data

Imputation of missing values for the eHLQ items was performed as missing data were numerous and following a missing at random pattern. Missing values mainly concerned older patients (median age = 71 vs. 62.5) with a lower level of health literacy (median BHLS of 8/15 vs. 10/15, median FCCHL score of 44/70 vs. 48/70). Imputed data were used to assess the psychometric parameters of the questionnaire (*N* = 371). We compared the factor structure derived from imputed versus complete-case datasets. Results were highly consistent (correlation 0.98 between imputed loadings and complete case loadings), indicating that the PCA-based imputation did not materially alter the psychometric findings (not presented). Spearman correlations between the proportion of missing responses and eHLQ dimension scores were weak (*r* < 0.20), suggesting limited risk of bias at the scale level.

### Item quality assessment

The results of the item quality assessment based on imputed data (*N* = 371) are shown in Table SUP4. Missing data ranged from 6.2% to 16.7% per item. Item Q31S7 had the most missing data (*n* = 62, 16.7%). There was no floor or ceiling effect (not presented). The higher the difficulty index, the “easier” the item, which means that most patients agreed or strongly agreed with the item. The highest score was for the scale 4 “Feel safe and in control” with a mean item difficulty of 3.1, while it was 2.6 for scale 1 “Using technology to process health information. Overall, the discrimination index was satisfactory for all items (> 0.40) [30], with the exception of item Q3S6, with a discrimination index of 0.31. For all but item Q3S6, Cronbach’s alpha declined in value when the item was removed.

### Construct validity

**Fig. 2:**
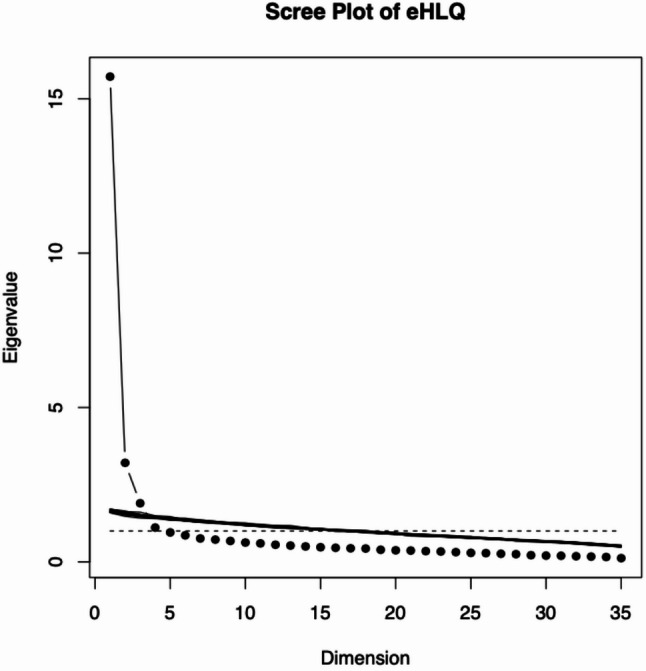
Scree plot of the eHLQ (imputed data, *N* = 371)

To assess the multiscale structure of the eHLQ, a scree plot with PCA-determined eigenvalues was used on imputed data (*N* = 371) (Fig. 2). The results showed that one component was significantly above the others with an eigenvalue of 15.7. Three components were above the parallel analysis, and 4 were above the threshold value of 1 (15.71, 3.21, 1.90 and 1.11).

The construct validity was assessed using imputed data (*N* = 371) and included the MTMM approach, which showed that items on scales 1, 3, 5, 6 and 7 were highly correlated (Figure SUP1) and the factor analysis below.

The Kaiser‒Meyer‒Olkin (KMO) measure of sampling adequacy was 0.9 [36], and Bartlett’s test of sphericity was significant (*p* < 0.001) [37], indicating that a factor analysis was suitable. A confirmatory factor analysis (CFA) based on the original 7-factor structure was initially conducted. However, the CFA yielded unstable estimates due to a non–positive definite covariance matrix of the latent variables, reflecting extremely high inter-factor correlations (S1–S3 = 0.91; S5–S6 = 0.99; S6–S7 = 0.99). These near-perfect correlations indicate substantial multicollinearity and construct redundancy, violating key assumptions of CFA and preventing meaningful model estimation. Under these conditions, CFA results could not be reliably interpreted. Therefore, an exploratory factor analysis (EFA) was undertaken to examine the underlying structure of the eHLQ in this population without imposing the original theoretical constraints.

The EFA was conducted using the unweighted least squares (ULS) estimation of factor loadings and an oblique-promax rotation [38]. Factor retention was based on a multiple decision rule combining the Kaiser criterion (eigenvalues > 1) [39], inspection of the scree plot, and parallel analysis. All three approaches converged toward a four-factor solution, which was therefore retained for interpretation as the most parsimonious and meaningful structure [40] (Table 3). Most items in scales 1 and 3 had loadings between 0.40 and 0.94 on the first factor. Items in scale 4 had loadings between 0.37 and 0.88 on the second factor. Most items in scales 5, 6 and 7 had loadings between 0.46 and 0.94 on the third factor. Items in scale 2 had loadings between 0.47 and 0.71 on the fourth factor. Five items showed cross-loadings (Q5S2, Q20S1, Q25S1, Q29S6 and Q12S2). The communalities were satisfactory (h2 > 0.40) [41] except for items Q3 (h2 = 0.37) and Q15 (h2 = 0.36). The correlation between rotated factor loadings obtained from the imputed and complete-case datasets was high, indicating strong stability of the factor structure across missing-data handling strategies.

**Table 3:** Rotated loadings of each item according to the 4-factor EFA with promax rotation in decreasing order of load (imputed data, *N* = 371)

Eigenvalues	Factor 1	Factor 2	Factor 3	Factor 4	h2^a^
	15.22	3.26	1.99	1.15	
Items					
Q6S3	**0.94**	0.05	-0.05	-0.02	0.83
Q7S1	**0.96**	-0.01	-0.09	-0.07	0.75
Q4S3	**0.87**	0.18	-0.01	-0.14	0.73
Q8S3	**0.86**	-0.02	-0.05	0.06	0.72
Q17S3	**0.74**	-0.03	-0.01	0.14	0.65
Q32S3	**0.69**	-0.04	-0.03	0.16	0.63
Q11S1	**0.61**	-0.04	0.23	-0.07	0.53
Q18S7	**0.50**	-0.11	0.28	0.21	0.65
Q5S2	**0.50**	0.02	-0.13	**0.38**	0.50
Q16S6	**0.44**	0.05	0.25	0.08	0.51
Q20S1	**0.40**	-0.06	**0.46**	-0.12	0.47
Q24S5	-0.17	-0.01	**0.94**	-0.16	0.57
Q28S7	0.05	-0.10	**0.82**	0.07	0.71
Q19S5	0.13	-0.02	**0.76**	-0.11	0.60
Q23S6	-0.09	0.07	**0.73**	-0.01	0.51
Q27S5	0.12	-0.13	**0.67**	0.13	0.60
Q13S1	0.12	0.06	**0.67**	-0.07	0.55
Q31S7	0.10	0.08	**0.62**	-0.01	0.53
Q34S6	0.22	0.01	**0.56**	0.10	0.62
Q33S7	0.28	0.10	**0.54**	-0.01	0.64
Q25S1	**0.41**	-0.03	**0.53**	-0.14	0.57
Q35S5	-0.15	0.15	**0.54**	0.20	0.48
Q9S6	0.27	-0.06	**0.53**	0.09	0.58
Q29S6	-0.26	0.15	**0.50**	**0.34**	0.53
Q2S5	0.09	0.18	**0.47**	-0.02	0.40
Q1S4	0.12	**0.88**	-0.08	-0.16	0.61
Q22S4	-0.13	**0.77**	0.11	0.01	0.65
Q3S6	0.14	**0.64**	-0.09	-0.06	0.37
Q10S4	-0.04	**0.63**	0.16	0.05	0.56
Q30S4	0.00	**0.58**	-0.03	0.27	0.58
Q14S4	-0.08	**0.37**	0.18	0.24	0.42
Q21S2	-0.01	0.13	-0.09	**0.71**	0.55
Q12S2	**0.31**	-0.00	-0.15	**0.65**	0.57
Q26S2	-0.02	-0.09	0.10	**0.64**	0.40
Q15S2	0.20	0.11	-0.11	**0.47**	0.36

^**a**^Communalities

Convergent and divergent validity assessment was performed on the raw data (*N* = 307), using Spearman’s correlation coefficients. There was a strong correlation (rho > 0.40, *p* < 0.001) between scale 2 of the eHLQ and the BHLS. The other scales showed a weak correlation with the BHLS and the FCCHL (rho < 0.40, *p* < 0.001), except for scale 3 (rho = 0.54 with the BHLS, *p* < 0.001). With regard to divergent validity, the HAQ score and eHLQ scores showed a weak negative correlation (-0.11 and − 0.21, respectively, *p* < 0.05 for scales 2 and 3).

All scales demonstrated satisfactory internal consistency, with Cronbach’s alpha values exceeding 0.78 and narrow confidence intervals. Estimates from McDonald’s omega was consistent with alpha values, supporting the robustness of the reliability findings (Table SUP5).

### Descriptive statistics

The sociodemographic characteristics show some notable differences (Table SUP6). Patients with higher education levels had higher scores on scales 1 “Using technology to process health information” with a median score of 2.8 (IQR: 2.2–2.3) versus 2.6 (IQR: 2.4-3.0), *p* = 0.01), on scale 2 “Understanding of health concepts and language” (median score of 3.0 (IQR: 3.0-3.4) versus 3.0 (IQR: 2.6-3,2), *p* < 0.001) and on scale 3 “Ability to actively engage with digital services” (3.0 (IQR: 2.4–3.4) versus 2.6 (IQR: 2.0–3.0), *p* = 0.01). Younger patients’ had significantly higher scores on scale 3 with a median score of 3.2 (IQR: 2.8–3.6) among patients under 45 years old compared with 2.5 (IQR: 2.0–3.0) among patients over 75 years (*p* < 0.001). Patients who lived alone scored lower on scale 1 with a median score of 2.4 (IQR: 2.0-2.8) than patients who lived with someone with 2.6 (IQR: 2.2-3.0) (*p* = 0.005).

## Discussion

A validated tool to measure the e-HL of French patients with CKD is necessary to develop appropriate digital health interventions for improving health equity in access to the kidney transplant waiting list. The eHLQ has shown satisfactory psychometric properties in different languages and populations [19–23].

In contrast to previous studies [19, 21–23], we did not conduct a confirmatory analysis. We encountered an issue with the covariance matrix of the latent variables, which was not positive definite due to extremely high correlations between certain scales. These near-perfect correlations indicate substantial redundancy between some factors, making the CFA results unstable. Therefore, EFA was conducted to explore the different dimensions of the eHLQ in a French population with CKD. Indeed, our results challenge the predefined structure of the eHLQ proposed by Kayser et al. [19]. Our work, based on the construct validity analysis, suggested a 4-scale structure that could be labelled as follows: “Ability to actively engage with digital services to access health information”, “Feel safe and in control”, “Use of digital services to interact with health professionals and to improve health” and “Understanding of health concepts”. This 4-scale structure, although coherent and meaningful, is questionable since the fourth factor was composed of only 4 items, one of which had cross-loadings (Q12S2) and one of which had a weak loading (Q15S2).

From a theoretical perspective, the differences observed in the factor structure of the eHLQ among patients with CKD may reflect the specific cognitive, clinical, and organisational context of this population. CKD is characterised by complex care trajectories involving multiple treatment options, frequent interactions with healthcare professionals, and increasing reliance on digital systems for monitoring, education, and care coordination. In such a context, patients may not clearly differentiate between cognitive processes (e.g. understanding health information) and behavioural capacities (e.g. engaging with digital services), as these competencies could be experienced as closely intertwined in everyday care. In addition, CKD is often associated with fatigue, cognitive burden, and emotional stress, which may further distort distinctions between theoretically separate dimensions of eHealth literacy. Rather than reflecting distinct latent constructs, eHealth literacy in this population may represent a more integrated and functional capability, oriented towards managing complex information within constrained cognitive and physical resources. This could partly explain why several original eHLQ dimensions appear highly correlated or merged in this sample [38, 46].

Regarding previous studies the best scores were found for the second scale (“Understanding of health concepts and language”) [19–22], whereas our study showed that among patients with CKD, the scale with the best score was the fourth (“Feel safe and in control”). These results suggest that our population is confident that health care data access management by providers is safe and controlled. In contrast, the worst scores were observed for scale 7 (“Digital services that suit individual needs”) in the Danish and Australian studies, whereas scale 6 (“Access to digital services that work”) was the worst in the Taiwanese study. In our study, the first scale, “Using technology to process health information”, was the scale that had lower patient agreement. These results may reflect a lower uptake of digital services in our population [42], which may have been influenced by the limited health literacy [5] and the older age of the population with CKD [43]. A lower level of education was also associated with a lower level of e-HL for scales 1 (“Using technology to process health information”) and 3 (“Ability to actively engage with digital services”), which reflected the appropriation of digital health services by individuals (Table SUP6).

Our study population was characterised by the proportion of older subjects (median age = 64 [53–72]) and the higher proportion of men than women (sex ratio M/F: 2/1), which was different from the Australian population (mean age = 56.8, sex ratio 0.63/1) [21]. Older age is associated with less use of digital services [43, 44], which could affect the perception of items [45]. For instance, the items in scale 7 (“Digital services that suit individual needs”) had the most missing data, which could be due to questionnaire completion fatigue (the items were in the last part of the eHLQ) or to the lower use of digital services; therefore, patients did not have a clear understanding of their needs with regard to digital health services. Unfortunately, we were not able to confirm this hypothesis because the information about the usage level of digital services was not collected.

The Cronbach’ reliability estimates were concordant in the different studies from Australia, Sweden, Taiwan and our study, between 0.75 and 0.95 for each scale. The lower estimate is found for the scale 2 for all the studies (0.75–0.82) and the higher for the scale 3 in Sweden (0.92) and France (0.93) which can reflect redundancy of items.

Our results were concordant with those of previous studies, as they reported strong correlations between scales 1, 3, and 5 and scales 6 and 7 [19, 21, 23]. To explain these correlations, they argued that these constructs could probably be located on the same causal path; consequently, the decision to keep the theoretical structure was made. However, our survey suggests that patients did not perceive these constructs as distinct in practice.

In France, patients with CKD have increasing access to digital health tools such as patient portals, teleconsultation platforms, and online educational resources. While these interventions aim to enhance patient engagement and access to information, their effectiveness largely depends on patients’ eHealth literacy. Digital health services are often developed without sufficient consideration of patients’ eHealth literacy levels, which may exacerbate the digital divide and unintentionally widen health disparities rather than reduce them [15, 47–49]. These findings highlight the need for digital interventions that are adapted to varying literacy levels and supported by healthcare professionals in order to promote equitable access to health information.

Although clinical outcomes were not assessed in this study, the results raise important questions about the relationship between eHealth literacy and health outcomes in patients with CKD. Limited eHealth literacy may hinder disease understanding, self-management, and engagement with care, potentially affecting access to complex pathways such as kidney transplantation, in the same way as the level of health literacy [6, 8]. Further longitudinal research is needed to explore these associations.

The eHLQ may serve as a useful tool to identify patients who could benefit from targeted support to improve their eHealth literacy. By identifying specific areas of difficulty, it could inform personalised digital or non-digital interventions, thereby promoting more equitable access to digital health resources for patients with CKD.

This study has several limitations that should be acknowledged. First, the single-centre design limits the generalisability of the factor structure to more diverse population, as the sample reflects a specific clinical context (old patients, high male proportion and low education level). The results should therefore be interpreted as exploratory and may not fully represent the diversity of experiences and eHealth literacy profiles observed in other settings or among patients with chronic kidney disease (CKD). Replication in larger and more heterogeneous samples is required to assess the robustness and transferability of the observed factor structure.

Second, the lack of data on patients’ actual digital service usage limits our ability to explore the relationship between eHLQ scores and digital service usage, which remains hypothetical.

Furthermore, although the exploratory factor analysis suggested a four-scale structure that appeared meaningful within this sample, these findings do not constitute definitive evidence for modification of the original eHLQ structure. Given the exploratory nature of the analyses, confirmatory factor analysis in an independent sample is necessary to formally test this alternative structure and to further evaluate its validity and reliability.

Most of the items in our study had numerous missing values, which raises questions about their understanding and relevance. Irrelevant items could have disrupted the preconceived structure of the eHLQ. Moreover, as the inter-item correlation is strong, particularly within the 3rd scale with a Cronbach’s above 0.90, item redundancy is presumed. Furthermore, some items are confusingly worded (Q13S1, Q25S1, Q4S3, Q14S4, Q2S5, Q3S6, Q23S6 and Q34S6), some have cross-loadings (Q5S2, Q20S1, Q25S1, Q29S6 and Q12S2), or loadings between 0.30 and 0.50 (Q16S6, Q20S1, Q2S5, Q14S4 and Q15S2), which calls for caution in their interpretation [33]. The systematic review and refinement of these items — including potential elimination, rewording, and theoretical re-evaluation within the eHLQ framework — will be conducted as part of the subsequent phase of this research programme.

Finally, limitations inherent in self-report studies and difficult to avoid are social desirability and recall bias, which can affect the respondent. Although caregiver-assisted questionnaire completion concerned only a minority of participants, this mode of administration may have introduced response bias, particularly among individuals with lower literacy levels. While this limitation could not be analytically addressed in the present study, it is acknowledged as a potential source of bias that may influence interpretation of the findings.

Importantly, a larger multicentre study involving diverse clinical settings with different demographic profiles, and standardised administration procedures is currently underway (data concerning the digital service usage is collected). This ongoing work will enable confirmatory validation, iterative refinement of the instrument, and assessment of its psychometric properties across different literacy strata, thereby strengthening external validity and supporting more robust conclusions regarding the applicability of the eHLQ in French patients with CKD.

## Conclusion

e-HL should be assessed using instruments adapted to the health literacy and eHealth literacy levels of patients with CKD in order to inform the development of appropriate digital health interventions and promote equitable access to information, health education, and healthcare. The present study provides an exploratory assessment of the eHLQ in French patients with CKD and suggests that a four-factor structure may better reflect patients’ perceived needs and abilities in this context. However, these findings should be interpreted cautiously and do not constitute definitive evidence for modification of the instrument’s structural architecture. Further iterative refinement, including a confirmatory validation in larger, multicentre cohorts of patients with CKD, is currently underway and will be necessary before a revised version of the eHLQ can be formally proposed.

## Electronic Supplementary Material

Below is the link to the electronic supplementary material.

Supplementary Material 1

Supplementary Material 2

Supplementary Material 3

Supplementary Material 4

Supplementary Material 5

Supplementary Material 6

Supplementary Material 7

Supplementary Material 8

## Data Availability

The datasets used and analysed during the current study are available from the corresponding author on reasonable request.

## Abbreviations

ESKD: End-stage kidney disease
CKD: Chronic kidney disease
HL: Health Literacy
e-HL: electronic health literacy
