# Supplementary material for: First assessment of the eHealth Literacy Questionnaire (eHLQ) among French people with chronic kidney disease
Source: BMC Nephrol. 2026 Jun 9;27:477. doi: 10.1186/s12882-026-04874-5 (PMC13479944; doi:10.1186/s12882-026-04874-5)
Supplement: Supplementary file 1 [file 12882_2026_4874_MOESM1_ESM.pdf]

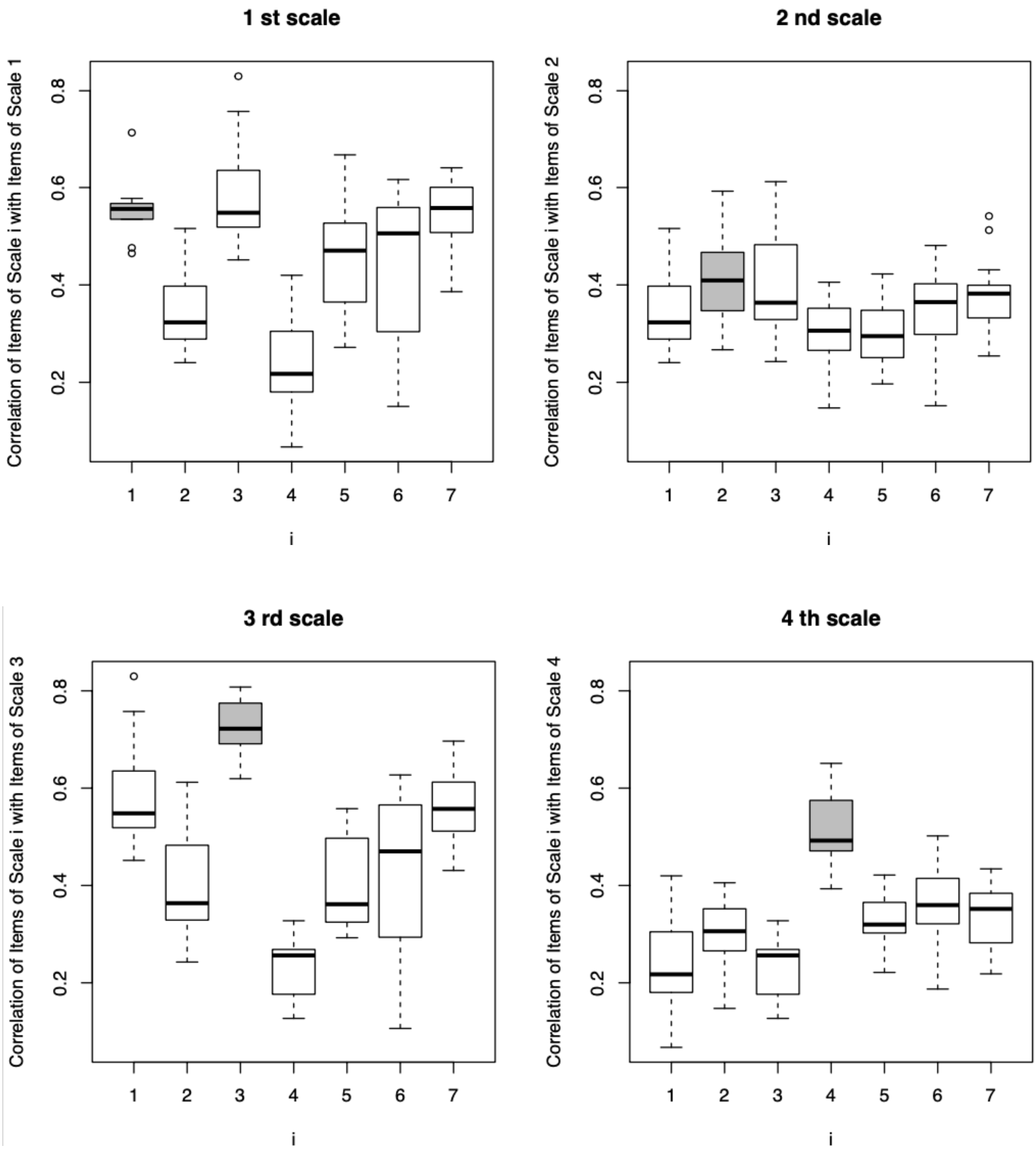
1 st scale
Correlation of Items of Scale i with Items of Scale 1
2 nd scale
Correlation of Items of Scale i with Items of Scale 2
3 rd scale
Correlation of Items of Scale i with Items of Scale 3
4 th scale
Correlation of Items of Scale i with Items of Scale 4
0.2
0.4
0.6
0.8
1
2
3
4
5
6
7
i

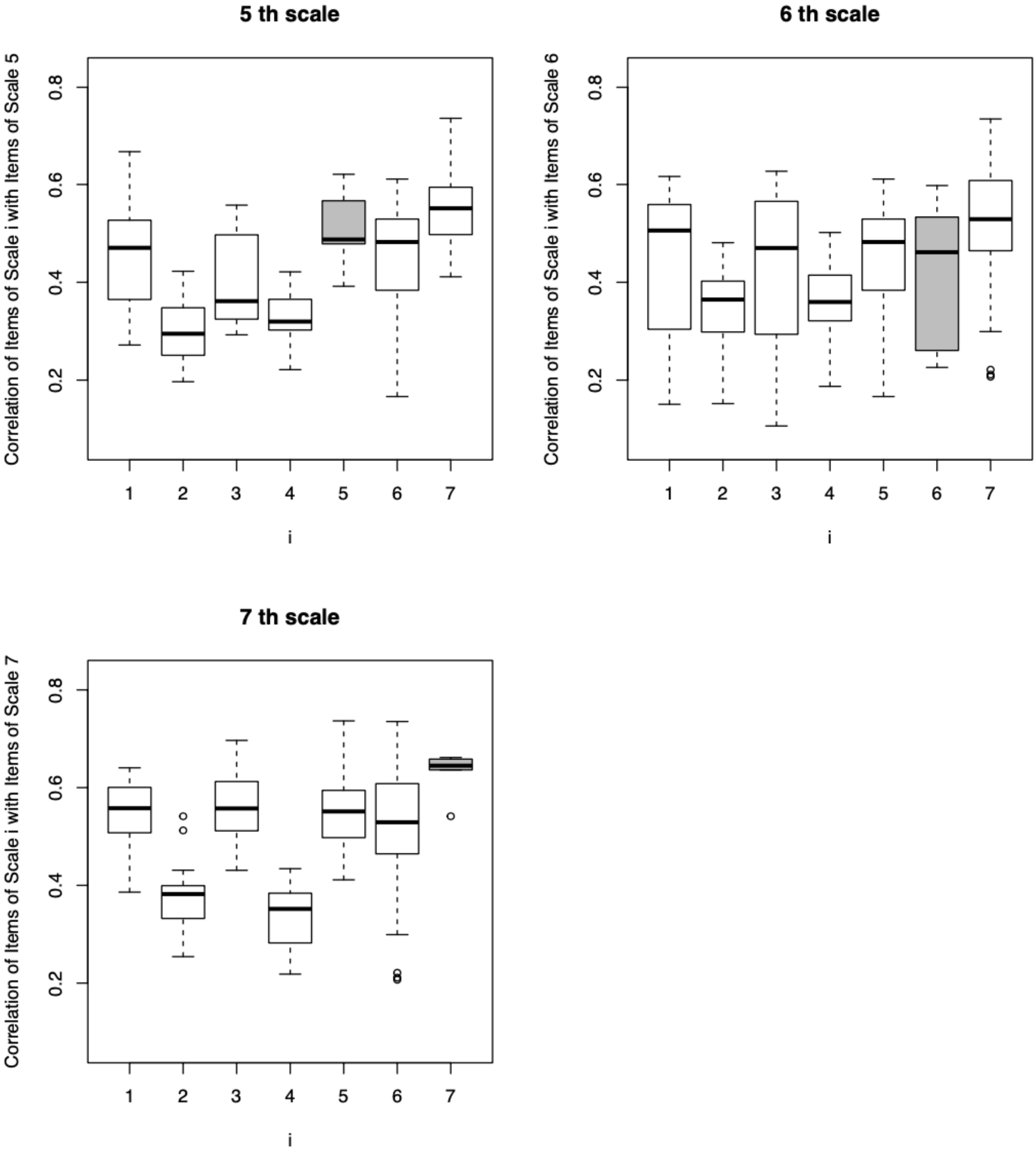

Fig. SUP1. MTMM approach, boxplots of inter-item correlations per scale (imputed data, N=371)
The grey boxes represent the corrected correlations of items with their own scale. The white boxes represent the correlations of items with the other scales. Grey boxes are supposed to be clearly above white boxes (meaning that item-total correlations are higher when the item belongs to its predefined scale)
