# Supplementary material for: First assessment of the eHealth Literacy Questionnaire (eHLQ) among French people with chronic kidney disease
Source: BMC Nephrol. 2026 Jun 9;27:477. doi: 10.1186/s12882-026-04874-5 (PMC13479944; doi:10.1186/s12882-026-04874-5)
Supplement: Supplementary file 2 [file 12882_2026_4874_MOESM2_ESM.pdf]

**Supplementary data - Index**

**Bartlett (Bartlett's sphericity test)**: Tests the null hypothesis that the correlation matrix is an identity matrix, i.e. that the variables in the data set are not correlated. If the test is significant, the null hypothesis is rejected: the correlation matrix is not an identity matrix and factor analysis is appropriate.

**Communality (h2)**: The proportion of common variance that a variable shares with other variables corresponds to communality. The communality value of a variable corresponds to the sum of the squares of the loadings between the variable and the factors (in EFA). Conversely, the unique variance of a variable corresponds to the portion of the total variance that is specific to that variable.

**Cronbach's alpha coefficient**: An estimator of internal consistency for a set of items, developed by Cronbach (1951). Its use requires three conditions (rarely met in practice): unidimensionality (the items measure a single factor), tau-equivalence (the true variance of the items is constant) and the absence of correlation between item errors. Its calculation is equivalent to dividing the data set in two in every possible way, then calculating the correlation coefficient for each division, and finally calculating the average of these values. It is considered acceptable if its value is greater than 0.70, but above 0.90 there is likely to be redundancy of items within the scale.

**Eigenvalue**: corresponds to the proportion of the overall variance in the data set explained by the common factor.

**Exploratory factor analysis (EFA):** The objective of EFA is to identify factors (or latent variables) that explain the common variance of the measured variables, excluding the specific variance (a single variable specific to each variable). The goal is to explain the maximum variance of the data set with the fewest possible factors and to identify these factors.

**Internal consistency**: Internal consistency (or internal coherence) is an aspect of the reliability of a measurement. It is a measure of the ability of items to measure the same concept. It assesses the degree of cohesion of a set of items intended to evaluate the same concept (or construct), which should theoretically be correlated.

**KMO (Kaiser-Meyer-Olkin) index**: Measure of sampling adequacy, a statistic indicating the proportion of variance in the variables in the data set that can be caused by underlying factors. This index takes values from 0 to 1. High values (close to 1) indicate that factor analysis may be useful. A KMO value greater than 0.80 is desirable, while a value less than 0.50 indicates that factor analysis is not appropriate.

**McDonald's omega coefficient**: Internal consistency estimator developed by McDonald (1978, 1985) based on a factorial analysis approach. This coefficient is reliable even when the tau-equivalence condition is not met.

**Parallel analysis:** An approach proposed by Horn (1965) to determine the number of latent variables to be extracted for exploratory factor analysis. The analysis consists of generating random uncorrelated data sets and comparing the eigenvalues obtained with the eigenvalues

of the data set under study. Only factors with eigenvalues significantly higher than the average random eigenvalues should be retained.

**Scree plot**: Diagram of eigenvalues as a function of factors (or principal components) based on principal component analysis (PCA) or exploratory factor analysis (EFA).
