# Supplementary material for: First assessment of the eHealth Literacy Questionnaire (eHLQ) among French people with chronic kidney disease
Source: BMC Nephrol. 2026 Jun 9;27:477. doi: 10.1186/s12882-026-04874-5 (PMC13479944; doi:10.1186/s12882-026-04874-5)
Supplement: Supplementary file 3 [file 12882_2026_4874_MOESM3_ESM.pdf]

1) How confident are you filling out medical forms by yourself?

Extremely	Quite a bit	Somewhat	A little bit	Not at all
☐	☐	☐	☐	☐

2) How often do you have someone help you read hospital materials?

All of the time	Most of the time	Some of the time	A little of the time	None of the time
☐	☐	☐	☐	☐

3) How often do you have problems learning about your medical condition because of difficulty understanding written information?

All of the time	Most of the time	Some of the time	A little of the time	None of the time
☐	☐	☐	☐	☐

Table SUP1. BHLS Brief health literacy screen
