# Supplementary material for: First assessment of the eHealth Literacy Questionnaire (eHLQ) among French people with chronic kidney disease
Source: BMC Nephrol. 2026 Jun 9;27:477. doi: 10.1186/s12882-026-04874-5 (PMC13479944; doi:10.1186/s12882-026-04874-5)
Supplement: Supplementary file 4 [file 12882_2026_4874_MOESM4_ESM.pdf]

When you receive a leaflet/brochure from your general practitioner/the hospital/the pharmacist, how often does it happen that:					
	Never	Rarely	Sometimes	Occasionally	Often
The letters are too small to read even when you are wearing glasses?	❑	❑	❑	❑	❑
There are words or signs that you do not understand?	❑	❑	❑	❑	❑
The content is too difficult to understand?	❑	❑	❑	❑	❑
It takes a long time to understand the content?	❑	❑	❑	❑	❑
You need help from others to understand the content?	❑	❑	❑	❑	❑

When you are searching for health information, how difficult is it for you to:					
	Never	Rarely	Sometimes	Occasionally	Often
Collect information about your illness via various ways (for example internet, encyclopedias, friends or family?	❑	❑	❑	❑	❑
To find exact information you are looking for?	❑	❑	❑	❑	❑
To share your thoughts about your illness with others? (for example, family, friends or healthcare providers?	❑	❑	❑	❑	❑
To understand the information you obtained?	❑	❑	❑	❑	❑
To use the information in daily life?	❑	❑	❑	❑	❑

When you are searching for health information, how difficult is it for you to:					
	Never	Rarely	Sometimes	Occasionally	Often
To judge whether the information applies to you?	❑	❑	❑	❑	❑
To check whether the information is correct and reliable?	❑	❑	❑	❑	❑
To judge whether the information is correct and reliable?	❑	❑	❑	❑	❑
To use the information to make decisions about your health? (like medication or daily activities?)	❑	❑	❑	❑	❑

Table SUP2. FCCHL (Functional, Communicative and Critical Health Literacy) scale
