# Supplementary material for: First assessment of the eHealth Literacy Questionnaire (eHLQ) among French people with chronic kidney disease
Source: BMC Nephrol. 2026 Jun 9;27:477. doi: 10.1186/s12882-026-04874-5 (PMC13479944; doi:10.1186/s12882-026-04874-5)
Supplement: Supplementary file 5 [file 12882_2026_4874_MOESM5_ESM.pdf]

In this section, we are interested in learning how your illness affects your ability to function in daily life.

Please check the response which best describes your usual abilities over the past week:

	Without ANY difficulty	With SOME difficulty	With MUCH difficulty	Unable to do
**DRESSING & GROOMING**<br>Are you able to:				
-Dress yourself, including tying shoelaces and doing buttons?	/___/	/___/	/___/	/___/
-Shampoo your hair?	/___/	/___/	/___/	/___/
**RISING**<br>Are you able to:				
-Stand up straight from a chair?	/___/	/___/	/___/	/___/
-Get in and out of bed?	/___/	/___/	/___/	/___/
**EATING**<br>Are you able to:				
-Cut your meat?	/___/	/___/	/___/	/___/
-Lift a full cup or glass to your mouth?	/___/	/___/	/___/	/___/
-Open a new milk carton?	/___/	/___/	/___/	/___/
**WALKING**<br>Are you able to:				
-Walk outdoors on flat ground?	/___/	/___/	/___/	/___/
-Climb up five steps?	/___/	/___/	/___/	/___/

Please check any aids or devices that you usually use for any of these activities:

Cane	/___/	Devices used for dressing (button hook, zipper pull, long-handled shoe horn, etc.)	/___/
Walker	/___/	Built up or special utensils	/___/
Crutches	/___/	Special or built-up chair	/___/
Wheelchair	/___/	Other (Specify: ………………………………)	/___/

Please check any categories for which you usually need help from another person:

Dressing or grooming	/___/	Eating	/___/
Rising	/___/	Walking	/___/

Please check the response which best describes your usual abilities over the past week:

	Without ANY difficulty	With SOME difficulty	With MUCH difficulty	Unable to do
**HYGIENE**<br>Are you able to:<br>-Wash and dry your body?<br>-Take a tub bath?<br>-Get on and off the toilet?	/___/<br>/___/<br>/___/	/___/<br>/___/<br>/___/	/___/<br>/___/<br>/___/	/___/<br>/___/<br>/___/
**REACH**<br>Are you able to:<br>-Reach and get down a 5-pound object (such as a bag of sugar) from just above your head?<br>-Bend down to pick up clothing from the floor?	/___/<br>/___/	/___/<br>/___/	/___/<br>/___/	/___/<br>/___/
**GRIP**<br>Are you able to:<br>-Open car doors?<br>-Open jars which have been previously opened?<br>-Turn faucets on and off?	/___/<br>/___/<br>/___/	/___/<br>/___/<br>/___/	/___/<br>/___/<br>/___/	/___/<br>/___/<br>/___/
**ACTIVITIES**<br>Are you able to:<br>-Run errands and shop?<br>-Get in and out of a car?<br>-Do chores such as vacuuming or yard work?	/___/<br>/___/<br>/___/	/___/<br>/___/<br>/___/	/___/<br>/___/<br>/___/	/___/<br>/___/<br>/___/

Please check any aids or devices that you usually use for any of these activities:

Raised toilet seat /___/

Bathtub seat /___/

Jar opener (for previously opened jars) /___/

Bathtub bar /___/

Long-handled appliances for reach /___/

Long-handled appliances in bathroom /___/

Other (Specify:...)

Please check any categories for which you usually need help from another person:

Hygiene /___/

Reach /___/

Gripping and opening things /___/

Errands and chores /___/

Table SUP3. HAQ health assessment questionnaire
