# Supplementary material for: First assessment of the eHealth Literacy Questionnaire (eHLQ) among French people with chronic kidney disease
Source: BMC Nephrol. 2026 Jun 9;27:477. doi: 10.1186/s12882-026-04874-5 (PMC13479944; doi:10.1186/s12882-026-04874-5)
Supplement: Supplementary file 6 [file 12882_2026_4874_MOESM6_ESM.pdf]

Items per scale	Item difficulty[a]	Discrimination index[b]	Cronbach's alpha	Acceptance (missing)[d]
**Scale 1.**	**2.60 (0.70)**		**0.86**	
Q7	2.70 (0.89)	0.71	0.83 [c]	8
Q11	2.50 (0.87)	0.75	0.82 [c]	10
Q13	2.60 (0.80)	0.62	0.85 [c]	11
Q20	2.40 (0.80)	0.64	0.84 [c]	11
Q25	2.60 (0.84)	0.69	0.83 [c]	12
**Scale 2.**	**3.00 (0.50)**		**0.78**	
Q5	3.00 (0.50)	0.52	0.74 [c]	9
Q12	3.00 (0.70)	0.68	0.69 [c]	8
Q15	3.00 (0.71)	0.49	0.76 [c]	9
Q21	3.10 (0.59)	0.57	0.73 [c]	8
Q26	3.10 (0.72)	0.50	0.75 [c]	8
**Scale 3.**	**2.70 (0. 80)**		**0.93**	
Q4	2.70 (0.94)	0.78	0.92 [c]	10
Q6	2.70 (0.91)	0.87	0.90 [c]	9
Q8	2.60 (0.92)	0.83	0.91 [c]	9
Q17	2.60 (0.87)	0.80	0.91 [c]	10
Q32	2.70 (0.81)	0.78	0.92 [c]	13
**Scale 4.**	**3.10 (0.53)**		**0.84**	
Q1	3.20 (0.68)	0.61	0.82 [c]	6
Q10	3.00 (0.74)	0.67	0.80 [c]	10
Q14	3.00 (0.62)	0.56	0.83 [c]	11
Q22	3.10 (0.70)	0.75	0.78 [c]	9
Q30	3.20 (0.58)	0.63	0.81 [c]	11
**Scale 5.**	**2.80 (0.58)**		**0.84**	
Q2	2.80 (0.77)	0.55	0.82 [c]	11
Q19	2.70 (0.75)	0.69	0.78 [c]	11
Q24	2.60 (0.79)	0.63	0.80 [c]	11
Q27	2.80 (0.75)	0.70	0.78 [c]	11
Q35	3.10 (0.66)	0.58	0.81 [c]	11
**Scale 6.**	**2.80 (0.55)**		**0.82**	
Q3	3.20 (0.64)	0.31	0.83 [c]	9
Q9	2.70 (0.84)	0.69	0.76 [c]	11
Q16	2.60 (0.84)	0.61	0.78 [c]	11
Q23	2.70 (0.74)	0.63	0.77 [c]	15
Q29	3.00 (0.67)	0.55	0.79 [c]	12
Q34	2.70 (0.77)	0.68	0.76 [c]	15
**Scale 7.**	**2.70 (0.66)**		**0.87**	
Q18	2.70 (0.81)	0.70	0.85 [c]	11
Q28	2.80 (0.73)	0.76	0.82 [c]	13
Q31	2.80 (0.72)	0.70	0.85 [c]	17
Q33	2.70 (0.75)	0.75	0.83 [c]	15

Table SUP4. Quality assessment of the items per scale (imputed data, N=371)

[a] mean (sd), from 1 to 4

[b] Corrected item-total correlation: correlation between an item and the total score excluding that item

[c] Cronbach's alpha if item deleted

[d] Percentage
