# Supplementary material for: First assessment of the eHealth Literacy Questionnaire (eHLQ) among French people with chronic kidney disease
Source: BMC Nephrol. 2026 Jun 9;27:477. doi: 10.1186/s12882-026-04874-5 (PMC13479944; doi:10.1186/s12882-026-04874-5)
Supplement: Supplementary file 7 [file 12882_2026_4874_MOESM7_ESM.pdf]

eHealth Literacy Questionnaire scales	Alpha coefficient[a]	Omega coefficient[a]
Scale 1. Using technology to process health information	0.86 [0.83-0.89]	0.87 [0.84-0.89]
Scale 2. Understanding of health concepts and language	0.78 [0.73-0.82]	0.78 [0.75-0.82]
Scale 3. Ability to actively engage with digital services	0.93 [0.91-0.94]	0.93 [0.92-0.94]
Scale 4. Feel safe and in control	0.84 [0.81-0.87]	0.85 [0.82-0.87]
Scale 5. Motivated to engage with digital services	0.83 [0.80-0.87]	0.84 [0.81-0.86]
Scale 6. Access to digital services that work	0.82 [0.78-0.85]	0.83 [0.80-0.86]
Scale 7. Digital services that suit individual needs	0.87 [0.84-0.90]	0.87 [0.85-0.89]

Table SUP5. Reliability estimates per scale (imputed data, N=371)
[a]Estimate [Confidence Interval 95%]
