# Supplementary material for: First assessment of the eHealth Literacy Questionnaire (eHLQ) among French people with chronic kidney disease
Source: BMC Nephrol. 2026 Jun 9;27:477. doi: 10.1186/s12882-026-04874-5 (PMC13479944; doi:10.1186/s12882-026-04874-5)
Supplement: Supplementary file 8 [file 12882_2026_4874_MOESM8_ESM.pdf]

Factors	N	Median S1 (Q1,Q3)	Test (p value)	Median S2 (Q1,Q3)	Test (p value)	Median S3 (Q1,Q3)	Test (p value)	Median S4 (Q1,Q3)	Test (p value)
**Gender**	370		13728[a]		14306[a]		13950[a]		14400[a]
**Male**	243	2.6 (2.2,3.0)	(0.32)	3.0 (2.8,3.4)	(0.24)	2.8 (2.0,3.2)	(0.33)	3.0 (2.8,3.6)	(0.053)
**Female**	127	2.6 (2.2,3.0)		3.0 (2.6,3.2)		2.8 (2.0,3.0)		3.0 (2.8,3.2)	
**Age**	358	2.6 (2.2,3.0)	-0.07[b] (0.19)	3.0 (2.8,3.3)	-0.07[b] (0.21)	2.8 (2.0,3.0)	-0.26[b] (<0.001)***	3.0 (2.8,3.4)	-0.07[b] (0.21)
**Education level**	360		10616[a]		9424[a]		10616[a]		13055[a]
**High school or below**	235	2.6 (2.2,3.0)	(0.01)**	3.0 (2.6,3.2)	(<0.001)***	2.6 (2.0,3.0)	(0.013)*	3.0 (2.8,3.4)	(0.63)
**Bachelor's degree or college education**	125	2.8 (2.4,3.0)		3.0 (3.0,3.4)		3.0 (2.4,3.4)		3.0 (2.8,3.4)	
**Employment status**	371		2.24[c]		4.09[c]		20.58[c]		8.80[c]
**Employed**	90	2.6 (2.2,3.0)	(0.69)	3.0 (2.8,3.4)	(0.39)	3.0 (2.6,3.4)	(<0.001)***	3.0 (2.8,3.4)	(0.07)
**Unemployed**	42	2.6 (2.4,3.0)		3.0 (2.7,3.6)		2.8 (2.3,3.0)		3.4 (3.0,3.8)	
**Student**	4	2.7 (1.8,3.5)		3.3 (3.0,3.7)		3.5 (3.3,3.7)		3.5 (2.9,3.8)	
**Retired**	207	2.6 (2.2,3.0)		3.0 (2.8,3.2)		2.6 (2.0,3.0)		3.0 (2.8,3.4)	
**Others**	28	2.5 (2.0,2.8)		3.0 (2.6,3.4)		2.5 (2.0,3.0)		3.2 (2.8,3.5)	
**Living area**	364		0.75[c]		4.33[c]		2.30[c]		1.89[c]
**City centre**	88	2.6 (2.2,3.0)	(0.69)	3.0 (2.8,3.4)	(0.11)	3.0 (2.1,3.4)	(0.32)	3.0 (2.8,3.6)	(0.39)
**Suburban area**	98	2.8 (2.2,3.0)		3.0 (2.6,3.2)		2.8 (2.0,3.0)		3.0 (2.8,3.4)	
**Countryside**	178	2.6 (2.2,3.0)		3.0 (2.8,3.4)		2.8 (2.0,3.0)		3.0 (2.8,3.4)	
**Living alone**	363		12050[a]		11434[a]		11539[a]		10160[a]
**Lives with someone**	274	2.6 (2.2,3.0)	(0.005)**	3.0 (2.8,3.4)	(0.12)	2.8 (2.2,3.2)	(0.06)	3.0 (2.8,3.4)	(0.9)
**Lives alone**	89	2.4 (2.0,2.8)		3.0 (2.6,3.2)		2.5 (2.0,3.0)		3.0 (2.8,3.4)	
**Treatment modality**	352		4.27[c]		2.22[c]		8.13[c]		1.08[c]
**CKD**	94	2.8 (2.3,3.0)	(0.51)	3.0 (2.8,3.4)	(0.53)	3.0 (2.4,3.2)	(0.04)	3.0 (2.8,3.4)	(0.78)
**Autonomous dialysis**	70	2.4 (2.2,3.0)		3.0 (2.8,3.2)		2.6 (2.0,3.0)		3.0 (2.8,3.6)	
**In-centre dialysis**	84	2.6 (2.0,3.0)		3.0 (2.8,3.4)		2.6 (2.0,3.0)		3.0 (2.8,3.4)	
**Kidney transplantation**	104	2.6 (2.2,3.0)		3.0 (2.8,3.4)		3.0 (2.3,3.2)		3.0 (2.8,3.4)	
**Transplantation status**	293		0.90[c]		5.80[c]		8.64[c]		1.49[c]
**Transplanted**	105	2.6 (2.2,3.0)	(0.49)	3.0 (2.8,3.4)	(0.33)	3.0 (2.4,3.2)	(0.12)	3.0 (2.8,3.4)	(0.91)
**Registered on the list**	50	2.6 (2.4,3.0)		3.0 (2.8,3.4)		2.8 (2.2,3.0)		3.2 (2.8,3.6)	
**Transplantation assessment**	22	2.4 (2.0,2.8)		3.0 (2.8,3.4)		2.6 (2.0,3.0)		3.1 (2.7,3.3)	
**Temporary contraindication**	12	2.4 (2.0,3.0)		3.2 (2.9,3.3)		2.4 (1.8,3.1)		3.0 (2.8,3.6)	
**Not registered on the list**	80	2.6 (2.0,3.0)		3.0 (2.6,3.2)		2.6 (2.2,3.2)		3.0 (2.8,3.4)	
**Does not feel concerned about transplantation**	24	2.4 (1.9,3.0)		3.2 (2.7,3.4)		2.4 (1.5,2.7)		3.0 (2.8,3.4)	

Factors	N	Median S5 (Q1,Q3)	Test (p value)	Median S6 (Q1,Q3)	Test (p value)	Median S7 (Q1,Q3)	Test (p value)
**Gender**	370		15403[a]		14475[a]		13188[a]
**Male**	243	2.8 (2.6,3.2)	(<0.001)***	2.8 (2.5,3.2)	(0.03)*	3.0 (2.5,3.0)	(0.1)
**Female**	127	2.8 (2.4,3.0)		2.8 (2.4,3.0)		2.8 (2.2,3.0)	
**Age**		2.8 (2.5,3.0)	0.08[b] (0.16)	2.8 (2.5,3.2)	-0.08[b] (0.17)	2.8 (2.3,3.0)	-0.10[b] (0.09)
**Education level**	360		12154[a]		12003[a]		10558
**High school or below**	235	2.8 (2.6,3.0)	(0.9)	2.8 (2.5,3.0)	(0.58)	2.8 (2.2,3.0)	(0.14)
**Bachelor's degree or college education**	125	2.8 (2.4,3.2)		2.8 (2.5,3.2)		3.0 (2.5,3.0)	
**Employment status**	371		9.47[c]		6.08[c]		7.10[c]
**Employed**	90	2.8 (2.4,3.0)	(0.05)*	2.8 (2.5,3.0)	(0.19)	3.0 (2.5,3.0)	(0.13)
**Unemployed**	42	3.0 (2.6,3.2)		3.0 (2.7,3.3)		3.0 (2.5,3.2)	
**Student**	4	3.0 (2.4,3.5)		3.4 (3.0,3.6)		3.0 (2.6,3.4)	
**Retired**	207	2.8 (2.6,3.0)		2.8 (2.5,3.0)		2.8 (2.2,3.0)	
**Others**	28	2.8 (2.2,3.0)		2.7 (2.4,3.0)		2.5 (2.0,3.0)	
**Living area**	364		0.34[c]		0.18[c]		0.50[c]
**City centre**	88	2.8 (2.4,3.0)	(0.84)	2.8 (2.5,3.2)	(0.92)	3.0 (2.2,3.0)	(0.78)
**Suburban area**	98	2.8 (2.6,3.0)		2.8 (2.5,3.0)		2.8 (2.2,3.0)	
**Countryside**	178	2.8 (2.6,3.0)		2.8 (2.5,3.2)		2.8 (2.5,3.0)	
**Living alone**	363		10438[a]		10864[a]		10238[a]
**Lives with someone**	274	2.8 (2.6,3.0)	(0.25)	2.8 (2.5,3.2)	(0.19)	3.0 (2.5,3.0)	(0.20)
**Lives alone**	89	2.8 (2.4,3.0)		2.7 (2.5,3.0)		2.8 (2.2,3.0)	
**Treatment modality**	352		1.07[c]		2.66[c]		2.74[c]
**CKD**	94	2.8 (2.6,3.0)	(0.78)	3.0 (2.6,3.2)	(0.45)	3.0 (2.5,3.0)	(0.43)
**Autonomous dialysis**	70	2.8 (2.4,3.0)		2.8 (2.5,3.0)		2.8 (2.5,3.0)	
**In-centre dialysis**	84	2.8 (2.6,3.0)		2.8 (2.3,3.0)		2.8 (2.2,3.0)	
**Kidney transplantation**	104	2.8 (2.4,3.0)		2.8 (2.5,3.1)		3.0 (2.6,3.2)	
**Transplantation status**	293		5.53[c]		3.54[c]		4.96[c]
**Transplanted**	105	2.8 (2.4,3.0)	(0.35)	2.8 (2.5,3.2)	(0.62)	3.0 (2.5,3.0)	(0.42)
**Registered on the list**	50	3.0 (2.7,3.2)		2.8 (2.4,3.2)		2.8 (2.5,3.0)	
**Transplantation assessment**	22	2.8 (2.4,2.8)		2.7 (2.5,3.0)		2.7 (2.2,3.0)	
**Temporary contraindication**	12	2.8 (2.4,3.1)		2.7 (2.4,3.0)		2.6 (2.3,3.2)	
**Not registered on the list**	80	2.8 (2.4,3.0)		2.8 (2.3,3.2)		2.8 (2.2,3.0)	
**Does not feel concerned about transplantation**	24	2.8 (2.6,3.0)		2.8 (2.2,3.0)		2.8 (2.3,3.0)	

[a] Mann–Whitney U test
[b] Spearman's correlational coefficient (rho)
[c] Kruskal–Wallis test
* p value ≤ 0.05, ** p value ≤ 0.01, *** p value ≤ 0.001

Table SUP6. Comparison of eHLQ scores for scales 1 to 7 among covariates
